# Psychometric Properties of a Questionnaire to Assess Perceptions of Corporal Expression in Future Spanish Teachers

**DOI:** 10.3390/ijerph19106150

**Published:** 2022-05-18

**Authors:** Jorge Rojo-Ramos, Santiago Gomez-Paniagua, María Mendoza-Muñoz, Jorge Carlos-Vivas, Ángel Acevedo-Duque, Elizabeth Emperatriz García-Salirrosas, José Carmelo Adsuar

**Affiliations:** 1 Health, Economy, Motricity and Education (HEME) Research Group, Faculty of Sport Sciences, University of Extremadura, 10003 Cáceres, Spain; jadssal@unex.es; 2 BioẼrgon Research Group, University of Extremadura, 10003 Cáceres, Spain; 3 Promoting a Healthy Society Research Group (PheSO), Faculty of Sport Sciences, University of Extremadura, 10003 Cáceres, Spain; mamendozam@unex.es (M.M.-M.); jorge.carlosvivas@gmail.com (J.C.-V.); 4 Public Policy Observatory, Universidad Autónoma de Chile, Santiago 7500912, Chile; angel.acevedo@uautonoma.cl; 5 Faculty of Management Science, Universidad Autónoma del Perú, Lima 15842, Peru; egarciasa@autonoma.edu.pe

**Keywords:** corporal expression, future teachers, validation, questionnaire

## Abstract

Corporal expression is a content that is forgotten by most educators but has been proved to bring numerous benefits to students of all ages. Teacher perceptions and beliefs play a fundamental role in the teaching-learning process, influencing students to a great extent. This study aims to present the factor structure and reliability of a questionnaire for the assessment of teachers’ perceptions about corporal expression. The sample consisted of 212 Spanish prospective teachers who completed the questionnaire to assess their readiness and appreciation about corporal expression. Exploratory and confirmatory factor analyses, as well as reliability testing, were carried out. The results showed a factor structure with 3 dimensions (pleasure, preference, and evaluation of corporal expression) composed of 23 items with good and excellent goodness-of-fit values and high reliability (Cronbach’s alpha = 0.71–0.93). Thus, the questionnaire can be considered a quick and easy-to-apply tool to analyze prospective teacher’s perceptions about their preparation to address their students’ corporal expression, allowing stakeholders to take actions to promote it.

## 1. Introduction

There has been a renaissance of interest in learning about student and teacher attitudes toward many aspects of education in recent decades. The motivation for the study arises from the belief that changing student attitudes can improve teaching quality because educators can obtain insight into how students feel and utilize that information to make decisions regarding curricular programs and teaching tactics. All attitudes have emotional, cognitive, and behavioral components and may rely more on one than the others [1]. It has been shown that positive attitudes regarding a subject matter help the learning environment and vice versa [2]. For several authors, knowing the internal processes of the students has influenced and helped improve their learning [3].

In this sense, the pedagogical instrument to develop the expressive dimension of movement is none other than corporal expression (CE). CE has been proposed as a teaching and learning process where the student recognizes his possibilities, exercises them in actions and sequences in a sensitive and conscious way, and accepts them as experiences that can be transferred to other situations [4]. There are three main aspects of the approach to teaching body expression [5]: (1) the organization of teaching should be aimed at making the learner aware of his or her inner world; (2) the discovery of one’s own possibilities of expression and creation brings with it an immediate need to give them meaning through movement; and (3) as a result of the experience of the inner world and its externalization through movement structures supported by different techniques, there is a natural and logical need to organize the incipient personal repertoire under a structure that gives it meaning and allows the ultimate goal of all creation: aesthetic pleasure and communication to others. However, the block of corporal expression, perhaps because of its characteristics, is the one that has the least presence in education programs [6].

In the classroom, teacher attitudes are crucial. Attitudes have an impact on how teachers connect with their pupils and how curricular decisions are made in the classroom [7], understanding that they have the ability and power to make important decisions that will affect their function and the output of their students [8]. The attitudes and ideas of pre-service teachers regarding education are formed long before they join college through observational learning in elementary and secondary classes. Thus, its study is indispensable to being able to implement these actions in the classroom at a later stage.

Whether or not to include CE content depends on a great number of factors. Numerous studies have evaluated the inclusion of corporal expression content in academic curriculum, especially in the areas of physical and musical education [9,10], claiming CE is not recognized by the majority of teachers and that it is the least-valued element of curriculum [11,12]. This, combined with the fact that corporal expression is provided with a lower credit load during their studies and in a single year [13], means that many prospective professionals are uninterested in teaching corporal-expression-related information during their lessons. Also, few teachers have prior experience in this field during their childhood or youth, generating a context of ignorance in relation to this pedagogical issue [14]. In addition, previous publications have pointed out the dissatisfaction of teachers with the training received in terms of CE [15,16] that, with a wide range of content within CE and its continuous growth, can be characterized as a negative influence [17].

Another aspect to be taken into account is the student perception of CE content [18]. Students, a priori, show little positive attitude towards corporal expression, mainly due to the frequency with which teachers incorporate content of corporal expression, as well as to the methodology used [19]. Numerous studies have also demonstrated that a segment of the student body, particularly female students, has a lower level of interest [20], as it depends to a large extent on the motivational climate they perceive [21]. Moreover, the social stereotypes of the students have an impact on this issue [13]. This means that the teacher must make an effort to present this type of content in a new way for the students.

Therefore, it is essential to analyze the attitudes of future teachers towards corporal expression since their ability to develop a good classroom climate and enjoyable tasks for students allows the achievement of CE content. In addition, the validation of a quick and easy-to-use instrument allows us to know the current status of teachers in training in terms of CE content and to not focus on the barriers or previous experiences that affect its inclusion in classrooms, enabling the modification of previous training so that these issues are transferred to the classroom in the best possible way and generating the numerous benefits mentioned above in students at all stages.

Thus, the aim of this study is to present the factor structure and reliability of a questionnaire [22] assessing perceptions of corporal expression in future teachers from Spain. Furthermore, investigating the psychometric features of this instrument helps us determine whether it is a legitimate and reliable tool for stakeholders to use in implementing EC-related initiatives.

## 2. Materials and Methods

### 2.1. Instruments

To define the sample, a sociodemographic 4-item questionnaire was created, comprising questions regarding sex, age, faculty to which they belong, and education specialty.

Also, a validated questionnaire [22] was used, which evaluated perceptions of CE and consisted of 32 items grouped into 4 factors: (1) evaluation of CE (14 items), (2) preference (7 items), (3) pleasure (6 items), and (4) teacher’s attitude (5 items). The items of dimension (1) referred to the general importance of CE for life as a whole; the items from dimension (2) evaluated the joint observation of attitude towards CE versus other parts of the content; those from dimension (3) focused on positive feelings that the student had in relation to CE; and those belonging to dimension (4) allowed the measurement of student perception of how the teacher facilitated or helped them to have a good attitude or motivation towards CE. The responses were based on a Likert scale of 1 (strongly disagree) to 5 (strongly agree). The indirect items were transposed before data analysis so that they corresponded to each of the characteristics listed above. The authors claimed a consistency value of 0.95 in the original paper [21], which focused on analyzing such perceptions in secondary school students with >0.70 for each of the four dimensions. Moreover, all items related with the last questionnaire dimension (5 items), “teacher’s attitude”, were deleted due to the current scale context, which was applied to a population of prospective teachers rather than to secondary school students.

### 2.2. Participants

A total of 212 future teachers (Master’s students) from public universities in Extremadura were included in the study (Spain), representing 48% of the total number of Master’s students in the different training modalities. Table 1 summarizes their attributes. The participants were chosen using a convenience sampling procedure that was not based on probability [23].

### 2.3. Procedure

To access the sample, the collaboration of professors from the corporal expression areas of the different faculties of the University of Extremadura was requested so that they could provide the students with the questionnaire and the informed consent form through the virtual classroom.

The students who agreed to collaborate with the study were able to access the study using the Google Form tool via a URL link. The data were collected between January and February 2022.

It was decided to use an online questionnaire because it allowed the responses to be stored in the same database, facilitated the distribution of the instrument, and generated a higher response rate, avoiding the loss of data [24].

### 2.4. Statistical Analysis

The exploratory analyses (EFAs) were carried out using a free statistical package, FACTOR v.10.10.02 (Rovira I Virgili University: Tarragona, Spain) [25], which considered the ordinal nature of the data gathered using a 5-choice Likert scale. The factor extraction was performed using a robust unweighted least squares (RULS) approach with Promin rotation [26], assuming a correlation between them [27]. A polychoric correlation matrix [28] was employed to account for the nature of the data, and the proper number of dimensions was determined through the use of parallel analysis [29]. A normalized direct oblimin was chosen as the rotation method for defining factor simplicity and structure once the number of dimensions was determined. As sampling adequacy metrics, the Kaiser–Meyer–Olkin (KMO) and Bartlett tests of sphericity were employed [30].

The confirmatory factor analysis was then carried out using the AMOS v.26.0.0 software package (IBM Corporation, Wexford, PA, USA). The elements with loads less than 0.60, crossloads more than 0.40, and communalities less than 0.30 were removed [31]. The following indices were used to evaluate the model’s goodness-of-fit: a chi-squared probability defining as adequate nonsignificant values (*p* > 0.05) [32]; a root mean square error of approximation (RMSEA) [33]; a root mean square of residuals (RMSR) [34]; a chi-square per degree of freedom ratio (CMIN/DF) [35]; a non-normed fit index (NFI) [36]; and a comparative fit index (CFI) [37]. Cronbach’s alpha coefficient and McDonald’s omega were also used as reliability indices for evaluating the questionnaire’s final structure [38,39].

## 3. Results

Three components related to explained variance based on eigenvalues [40] and the reliability of expected a posteriori (EAP) scores [41] were provided using a RULS technique [42] with Promin rotation. Prior to the EFAs, four items (13, 15, 16, and 19) were eliminated due to values below 0.50 on the normed measure of sampling adequacy (MSA) [43]. Because of the good results provided by the sample adequacy indices (Bartlett test = 2331.4; df = 253; *p* = 0.000; and KMO test = 0.80419), the EFAs were carried out. After determining the number of dimensions, a normalized direct oblimin rotation method was chosen since the amount of kurtosis (kurtosis = 38.082; *p* = 0.000) necessitated non-parametric approaches. Table 2 reflects the rotated loading matrix for 23 items and 3 factors.

Observing the rotated loading matrix, we can see that there are 23 items, all of them with loadings higher than 0.3 and distributed in the three factors initially mentioned.

Table 3 shows the polychoric correlation matrix obtained in the exploratory analysis.

Table 3 shows the correlation between the ordinal variables (items) and items that showed values >0.3 with some type of relationship between them, with the highest scores being those that belonged to the same factor.

Each item’s structure and factor loadings are shown in Table 4 (Spanish version can be found in Appendix A), with three correlated factors composing the factorial solution.

Table 4 shows the loadings of the items above 0.3, which allowed us to assess which of them belonged to which factor.

Table 5 displays the correlation between the factors of the questionnaire.

Table 5 shows the correlation between factors 1 and 2, as well as 1 and 3, as the values exceeded the threshold of 0.3.

**Figure 1:**
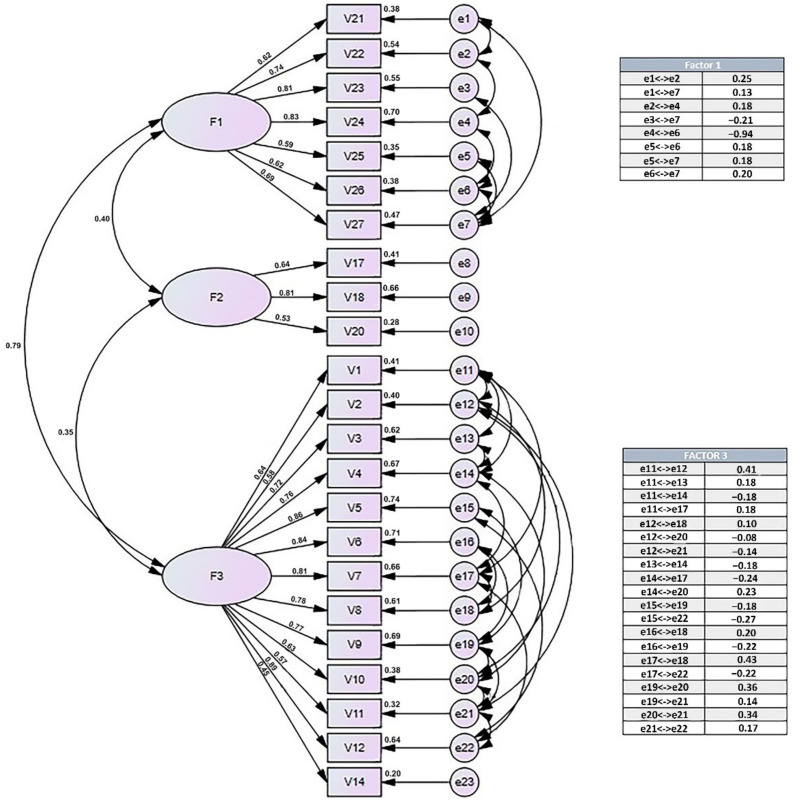
Questionnaire factor model.

The CFAs were used to build a definite model after the scheme of the questionnaire was set (Figure 1).

Figure 1 represents the final structure of the questionnaire composed of 23 items divided into 3 factors, showing from left to right the following values: (1) correlation between factors; (2) standardized regression weights; (3) squared multiple correlations of each variable; and (4) correlations between exogenous variables (tables).

The CEFI-R goodness-of-fit indices following the CFAs are shown in Table 6 [44]. They all showed a good match between the data and the model [45]. The chi-squared probability was great due to the nonsignificant values. Also, the RMSEA was within the prescribed limits (0.010–0.050), while the RMSR, being less than 0.08, could be classified as correct. In addition, given that it must be less than 2 for a valid model fit, the CMIN/DF index had great values. A close fit to the model was shown by NNFI and CFI values greater than 0.9.

Cronbach’s alpha, McDonald’s omega, and the explained variance of each factor were used to calculate reliability indices for the CEFI-R questionnaire dimensions in Table 7.

The Cronbach’s alpha and McDonald’s omega scores were satisfactory for each of the factors, as they were higher than 0.7 [46]. The explained variance represents the percentage of variance in the responses attributed to each of the factors of the model that was not attributed to hazard (residual values).

## 4. Discussion

The current study’s main contribution was an examination of the questionnaire’s psychometric properties to assess perceptions of corporal expression, as well as validity and reliability indicators for the questionnaire, in a sample of Spanish future teachers. The findings revealed a factor structure consisting of 3 connected dimensions with 23 items and optimal goodness-of-fit indices. Furthermore, the Cronbach’s alpha and McDonald’s omega values revealed a high level of consistency. Initially, the scale was made up of 4 factors and 32 items; however; it was applied in a physical education class and a secondary education student context. Thus, this research defined a short and useful tool to evaluate future teacher’s perceptions regarding CE, enabling university instructors to adapt their teaching and highlighting the importance of this content.

The first factor, “pleasure”, referred to positive feelings that the student had in relation to CE, and it was composed of seven items and found very good results for all of them. These questions were highly related to the previous experience of the sample in relation to the content of CE, so it must consider that the training profile would certainly influence the perception of these future teachers. For example, an earlier study found a correlation between the positive valuation of this content and previous dance experiences in Sport Sciences degree students [47]. It was also stated that not imparting or imparting CE with a lack of understanding had a negative impact on students’ attitudes toward it [48].

The next factor, “preference”, which involved the joint observation of the attitude towards CE versus other parts of the content, showed intermediate values, and it was composed of three items. In this sense, prior research showed that women had more positive attitudes than the opposite gender towards this content due to their previous experiences, valuing their effects to a greater extent [49,50]. Nevertheless, it should be mentioned that both genders showed interest in different styles of dance, revealing differences in attitudes towards school dance [51].

The last factor, “evaluation of CE”, as the general importance of CE for life, consisted of 12 items and reflected high values. Teachers have a significant impact on students’ attitudes and motivations, to the point that the “teacher” factor has been identified as one of the most important variables in the formation of favorable or unfavorable attitudes [52,53]. Moreover, the use of an appropriate structuration of CE content inside classes improved the student experience of pleasurable emotions, and this, in turn, improved the student attitude toward it [54]. Also, Rady and Schmidt highlighted the importance of educators stepping up their efforts to make artistic activities a viable alternative to competitive activities, particularly in order to extend the range of options available to all pupils [55].

There were various limitations to this study. The sample size was constrained. This research did not use direct data collection methods, such as face-to-face interviews, that present more valid and reliable results than telephone or online surveys [56]. This was preliminary work, as instrument validation is a process that takes time to develop. Because all of the subjects studied in the Extremadura region, sociocultural variables may have influenced the outcomes. In future research areas, it would be interesting to recruit a larger sample from other locations in Spain to gather further evidence on the questionnaire’s strengths.

## 5. Conclusions

The present study examined the validity and reliability of the questionnaire, which was used to assess future teachers’ perceptions towards corporal expression. Our findings revealed that a solution made up of 23 items and explained by 3 components had consistent goodness-of-fit indications, as well as good and outstanding reliability ratings. This questionnaire is appropriate for instructional and research purposes in educational institutions, and it is a free and simple-to-use tool that takes no more than five minutes to administer.

Analyzing teachers’ perceptions about CE in their training is critical because it affects their attitudes and self-efficacy, as well as their future labor and their ability to include this content in educational curriculum.

## Figures and Tables

**Table 1:** Sociodemographic characteristics of the sample (*n* = 212).

Variables	Categories	*n*	%
Sex	Men	46	21.7
	Women	166	78.3
Age	Under 20	21	9.9
	Between 20 and 30	184	86.8
	Between 31 and 40	4	1.9
	Over 40	3	1.4
Faculty	Teacher Training Faculty	182	85.8
	Faculty of Education and Psychology	22	10.4
	Faculty of Sports Sciences	8	3.8
Education Specialty	Early Childhood	146	68.9
	Primary School	66	31.1

**Table 2:** Rotated loading matrix with normalized oblimin.

Item	Factor 1	Factor 2	Factor 3
1. Corporal Expression is useful in teacher training.	0.000	−0.131	0.790
2. Corporal Expression allows to express feelings.	0.040	−0.46	0.729
3. The learning received in Corporal Expression is necessary and important.	0.105	−0.039	0.723
4. Corporal Expression classes improve the mood.	0.234	0.094	0.618
5. Corporal Expression helps to know oneself better, to relate to others, and to be creative.	0.042	0.037	0.883
6. Corporal Expression contributes to global education.	0.213	−0.065	0.758
7. Corporal Expression is good for socialization.	−0.065	0.070	0.924
8. Corporal Expression is a good social experience and gives you opportunities to get to know your peers in a deeper way.	−0.136	−0.023	1.017
9. In the Corporal Expression classes, a very positive environment is created.	0.138	0.110	0.706
10. Corporal Expression provides important relief from accumulated stress.	0.063	−0.008	0.710
11. Corporal Expression also improves overall health and not only physical fitness activities.	−0.065	0.051	0.743
12. The activities taught in Corporal Expression seem important to me.	0.205	−0.003	0.692
13. What is learned in Corporal Expression is useless.	Deleted		
14. I like Corporal Expression because it works on aesthetics and social relations.	0.121	0.217	0.367
15. I don’t like Corporal Expression because it doesn’t have as much risk or as many challenges as sports.	Deleted		
16. I find Corporal Expression interesting because it is not competitive.	Deleted		
17. I prefer Corporal Expression to other content.	−0.054	0.731	0.022
18. I prefer Corporal Expression because students interact with their peers more than when doing other motor skills content.	−0.011	0.671	0.214
19. Corporal Expression is not as fun as other content.	Deleted		
20. Corporal Expression is more important than the rest of the content.	0.105	0.638	−0.162
21. If doing Corporal Expression in classes were optional, I would choose to do it.	0.736	0.053	−0.051
22. When I have taken Corporal Expression classes, I have liked them because it is something different from what is normally taught.	0.918	−0.098	−0.051
23. When I have taken Corporal Expression classes, I have liked them because it is cooperative.	0.805	−0.061	0.088
24. When I have taken Corporal Expression classes, I have enjoyed the time I have spent doing these activities.	0.797	−0.095	0.174
25. When I have taken Corporal Expression classes, I have liked them because they include artistic activities.	0.571	0.078	0.113
26. When I have taken Corporal Expression classes, I have liked them because they involve more games.	0.577	0.183	0.062
27. When I have taken Corporal Expression classes (in my teacher training), I have always wanted more.	0.614	0.165	0.118

Note: These items are a literal translation into English for ease of reading, not a cross-cultural adaptation into English.

**Table 3:** Polychoric correlation matrix.

Item	1	2	3	4	5	6	7	8	9	10	11	12	14	17	18	20	21	22	23	24	25	26	27
1	1.00																						
2	0.79	1.00																					
3	0.68	0.63	1.00																				
4	0.52	0.57	0.61	1.00																			
5	0.73	0.71	0.76	0.80	1.00																		
6	0.69	0.70	0.75	0.72	0.82	1.00																	
7	0.73	0.69	0.67	0.65	0.83	0.79	1.00																
8	0.65	0.69	0.67	0.68	0.82	0.83	0.89	1.00															
9	0.56	0.55	0.62	0.71	0.73	0.67	0.76	0.74	1.00														
10	0.46	0.43	0.54	0.73	0.68	0.61	0.60	0.64	0.78	1.00													
11	0.43	0.36	0.51	0.62	0.57	0.61	0.60	0.65	0.66	0.72	1.00												
12	0.57	0.56	0.72	0.69	0.72	0.80	0.70	0.77	0.73	0.65	0.70	1.00											
14	0.34	0.37	0.33	0.49	0.53	0.46	0.44	0.41	0.39	0.43	0.38	0.40	1.00										
17	0.07	0.15	0.15	0.19	0.12	0.11	0.17	0.12	0.21	−0.01	0.03	0.09	0.29	1.00									
18	0.22	0.28	0.27	0.40	0.39	0.28	0.29	0.23	0.36	0.30	0.28	0.32	0.25	0.57	1.00								
20	−0.06	−0.02	0.05	0.10	0.05	0.05	0.09	0.01	0.12	0.02	0.07	0.14	0.16	0.42	0.46	1.00							
21	0.40	0.39	0.38	0.50	0.52	0.48	0.46	0.36	0.46	0.33	0.26	0.50	0.28	0.19	0.31	0.21	1.00						
22	0.45	0.46	0.54	0.58	0.58	0.57	0.48	0.46	0.54	0.44	0.33	0.55	0.37	0.17	0.30	0.07	0.67	1.00					
23	0.50	0.53	0.53	0.62	0.58	0.70	0.53	0.54	0.57	0.11	0.42	0.64	0.40	0.15	0.35	0.13	0.58	0.72	1.00				
24	0.60	0.57	0.65	0.66	0.64	0.67	0.57	0.60	0.68	0.58	0.50	0.68	0.36	0.13	0.33	0.15	0.59	0.76	0.82	1.00			
25	0.27	0.34	0.38	0.52	0.47	0.58	0.47	0.44	0.54	0.51	0.40	0.54	0.41	0.20	0.21	0.17	0.42	0.51	0.56	0.59	1.00		
26	0.32	0.40	0.34	0.44	0.48	0.50	0.56	0.44	0.49	0.36	0.38	0.45	0.38	0.26	0.36	0.22	0.47	0.55	0.57	0.57	0.56	1.00	
27	0.33	0.36	0.52	0.63	0.58	0.59	0.54	0.51	0.58	0.50	0.41	0.52	0.42	0.22	0.28	0.32	0.59	0.57	0.54	0.64	0.59	0.62	1.00

**Table 4:** Rotated factor solution and factor loadings.

Item	Factor 1	Factor 2	Factor 3
1. Corporal Expression is useful in teacher training.			0.790
2. Corporal Expression allows to express feelings.			0.729
3. The learning received in Corporal Expression is necessary and important.			0.723
4. Corporal Expression classes improve the mood.			0.618
5. Corporal Expression helps to know oneself better, to relate to others, and to be creative.			0.883
6. Corporal Expression contributes to global education.			0.758
7. Corporal Expression is good for socialization.			0.924
8. Corporal Expression is a good social experience and gives you opportunities to get to know your peers in a deeper way.			1.017
9. In the Corporal Expression classes, a very positive environment is created.			0.706
10. Corporal Expression provides important relief from accumulated stress.			0.710
11. Corporal Expression also improves overall health and not only physical fitness activities.			0.743
12. The activities taught in Corporal Expression seem important to me.			0.692
13. What is learned in Corporal Expression is useless.	Deleted		
14. I like Corporal Expression because it works on aesthetics and social relations.			0.367
15. I don’t like Corporal Expression because it doesn’t have as much risk or as many challenges as sports.	Deleted		
16. I find Corporal Expression interesting because it is not competitive.	Deleted		
17. I prefer Corporal Expression to other content.		0.731	
18. I prefer Corporal Expression because students interact with their peers more than when doing other motor skills contents.		0.671	
19. Corporal Expression is not as fun as other content.	Deleted		
20. Corporal Expression is more important than the rest of the content.		0.638	
21. If doing Corporal Expression in the classes were optional, I would choose to do it.	0.736		
22. When I have taken Corporal Expression classes, I have liked them because it is something different from what is normally taught.	0.918		
23. When I have taken Corporal Expression classes, I have liked them because it is cooperative.	0.805		
24. When I have taken Corporal Expression classes, I have enjoyed the time I have spent doing these activities.	0.797		
25. When I have taken Corporal Expression classes, I have liked them because they include artistic activities.	0.571		
26. When I have taken Corporal Expression classes, I have liked them because they involve more games.	0.577		
27. When I have taken Corporal Expression classes (in my teacher training), I have always wanted more.	0.614		

**Table 5:** Inter-factor correlation matrix.

Factors	Factor 1	Factor 2	Factor 3
Factor 1	1.000		
Factor 2	0.362	1.000	
Factor 3	0.710	0.215	1.000

**Table 6:** Goodness of fit indices.

Indices	Value
Ρ (*χ*^2^)	0.996
RMSEA	0.048
RMSR	0.041
CMIN/DF	1.481
NNFI	0.904
CFI	0.966

**Table 7:** Internal consistency parameters for the questionnaire.

Parameters	Factor 1	Factor 2	Factor 3
Cronbach’s alpha	0.873	0.713	0.927
McDonald’s omega	0.884	0.721	0.944
Explained variance	4.561	1.674	8.229

## Data Availability

The datasets used during the current study are available from the corresponding author on reasonable request.

## Appendix A

**Table A1:** Cuestionario para la Evaluación de la medida de las actitudes hacia la Expresión Corporal. Reprinted from ref. [22].

1. La Expresión Corporal es útil para mi formación.
2. La Expresión Corporal permite expresar mis sentimientos.
3. Los aprendizajes que recibo en Expresión Corporal son necesarios e importantes.
4. Las clases de Expresión Corporal mejoran mi estado de ánimo.
5. La Expresión Corporal me ayuda a conocerme mejor, relacionarme con los demás, y a ser creativo.
6. La Expresión Corporal contribuye a mi educación global.
7. La Expresión Corporal es buena para socializarse.
8. La Expresión Corporal es una experiencia social y te da oportunidades para conocer a tus compañeros de una manera más profunda.
9. En las clases de Expresión Corporal se crean ambientes muy positivos.
10. La Expresión Corporal me proporciona un importante alivio del estrés acumulado.
11. La Expresión Corporal también mejora tu salud a nivel general y no sólo las actividades de condición física.
12. Las actividades que aprendo en Expresión Corporal me parecen importantes.
17. Prefiero la Expresión Corporal a otros contenidos.
18. Prefiero la Expresión Corporal porque me relaciono con mis compañeros/as más que cuando hacemos otros contenidos.
20. La Expresión Corporal es más importante que el resto de los contenidos.
21. Si hacer expresión corporal en las clases de educación física fuera opcional, elegiría hacerla.
22. La Expresión Corporal me gusta porque es algo distinto a lo que hacemos normalmente.
23. Me gusta la Expresión Corporal porque es cooperativa.
24. Me gusta el tiempo que paso haciendo actividades de Expresión Corporal.
25. La Expresión Corporal me gusta porque en ella se hacen actividades artísticas.
26. La Expresión Corporal me gusta porque en ella hacemos más juegos.
27. Cuando acabo la clase de Expresión Corporal en Educación Física, me quedo con ganas de más.
